# Stenosing Tenosynovitis Following Ankle Fracture Causing Progressive Acquired Tibialis Posterior Dysfunction: A Case Report

**DOI:** 10.7759/cureus.63472

**Published:** 2024-06-29

**Authors:** Aysha Rajeev, George Koshy, Saurav Krishnan, Kailash Devalia

**Affiliations:** 1 Trauma and Orthopaedics, Gateshead Health NHS Foundation Trust, Gateshead, GBR; 2 General Medicine, Gateshead Health NHS Foundation Trust, Gateshead, GBR

**Keywords:** stenosing tenosinovitis, tibialis posterior, foot and ankle fracture, tenosynovitis, stenosing

## Abstract

Stenosing tenosynovitis of the ankle with osseous bone formation following an open reduction and internal fixation of the ankle is a rare clinical condition. We report a case of adult-acquired flat foot following an open reduction and internal fixation of a bi-malleolar fracture due to tibialis posterior tendon dysfunction caused by stenosing tenosynovitis. This was managed by open excision of the bony tunnel and debridement, along with calcaneal osteotomy and distalization of the tendon, resulting in good functional outcomes.

## Introduction

Stenosing tenosynovitis of the tibialis posterior tendon (TPT) was first described by Kulowski in 1936 [1]. This condition causes inflammation, thickening of the tendon sheath, fibrosis of the tendon with or without rupture, and reactive hypertrophy of the bone at the posterior malleolar groove [2]. Chronic tenosynovitis can lead to new bone formation and entrapment of the TPT, causing tendon dysfunction [3].

Tibialis posterior tendon dysfunction (TPTD) is an acquired, progressive, and degenerative condition of the foot and ankle caused by insufficiency of the tibialis posterior musculo-tendinous unit, leading to pain, stiffness, and weakness [4,5]. It can be secondary to an ankle fracture [6], ankle sprain [7], direct blow to the tendon [8], chronic tenosynovitis (due to traumatic, degenerative, or inflammatory arthritis), or due to an accessory navicular/tarsal coalition.

We describe a rare case of tibialis posterior tendon dysfunction caused by stenosis due to heterotopic new bone formation in the retro-malleolar groove following an open reduction and internal fixation for a bi-malleolar ankle fracture, with a successful outcome following surgical decompression and distalization of the tibialis posterior tendon.

## Case presentation

**Figure 1:**
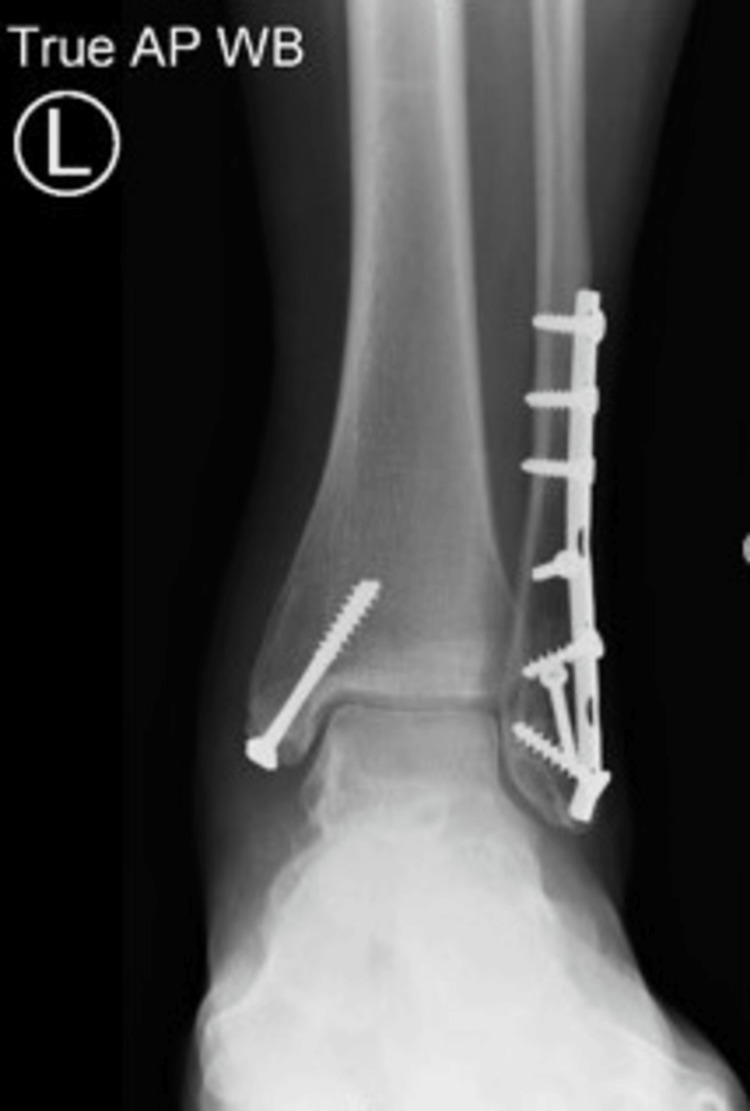
AP X-ray view of a bi-malleolar fracture treated with plate and screws showing good bony healing AP: anteroposterior

**Figure 2:**
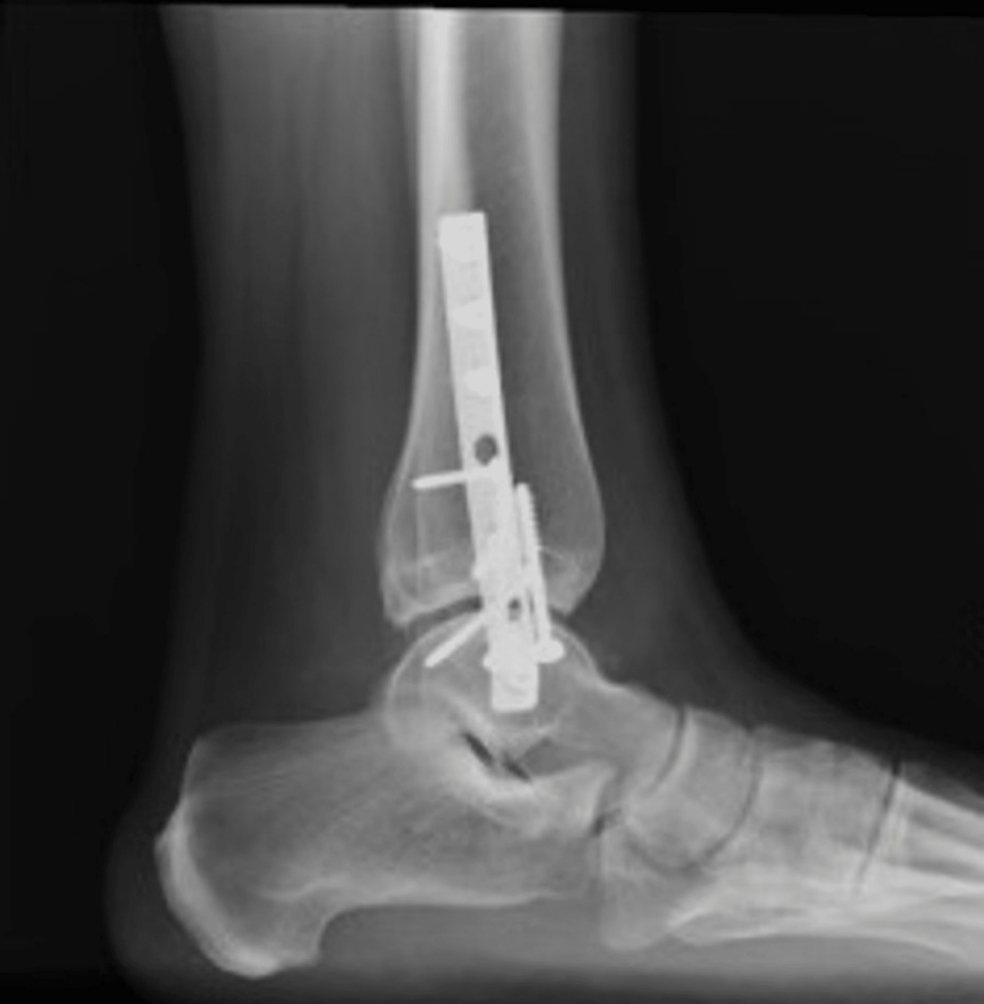
Lateral X-ray view of the bi-malleolar fracture treated with plate and screws showing good bony healing

A 51-year-old woman was referred to our Foot and Ankle clinic by her general practitioner after having undergone a bi-malleolar ankle fracture fixation about four years ago. Her presenting complaints were instability in the context of proprioceptive abnormality and pain associated with the medial malleolus and the tibialis posterior tendon on the left side. She experienced constant pain around the inside of her ankle and occasional sensations of unsteadiness but without actual episodes of the ankle giving way. On examination, she localized the tenderness to the medial malleolus and seemed to have tenderness associated with the screws. The tibialis posterior resistive examination revealed some discomfort and tenderness with no deformity, absent "too many toes" sign, and mild weakness on the heel raise test. Her ankle range of motion was slightly restricted, and she did not have any pain associated with the lateral metalwork. X-ray examination (Figures 1-2) showed the fracture was well united with no evidence of ankle arthrosis or malunion.

An ultrasound scan revealed some scarring and thickening around the tibialis posterior tendon as well as inflammatory changes. The patient underwent removal of the metalwork to reduce tendon irritation and relieve pain. However, she returned to the clinic after one year complaining of increasing pain, especially along the tibialis posterior tendon. Clinical examination at that stage revealed moderate swelling and tenderness along the tibialis posterior tendon with a flexible deformity. The heel raise test showed moderate weakness, and the "too many toes" sign was positive. She had progressed from stage 1 to stage 3 according to the Johnson & Strom classification of adult-acquired flatfoot (Table 1).

**Table 1:** Classification of Johnson & Strom adult-acquired flat foot Modified from [9]

Variable	Stage 1: Mild, medial pain	Stage 11: Moderate, medial pain	Stage 111: Severe, medial pain
Examination swelling and tenderness	Mild swelling and tenderness along the tibialis posterior tendon	Moderate swelling and tenderness along the tibialis posterior tendon	No swelling but marked tenderness in the tibialis posterior tendon
Heel raise test	Mild weakness	Moderate weakness	Marked weakness
“Too many toes” sign	Absent	Present	Present
Deformity	Absent	Present (flexible)	Present (fixed)
Pathologic features	Normal tendon length, para-tendinitis	Elongated and longitudinal tears	Disrupted with visible tears
Images	No changes	Gross deformity	Deformity with arthritis
Treatment	Conservative, tenosynovectomy	Flexor hallucis tendon transfer	Triple arthrodesis

**Figure 3:**
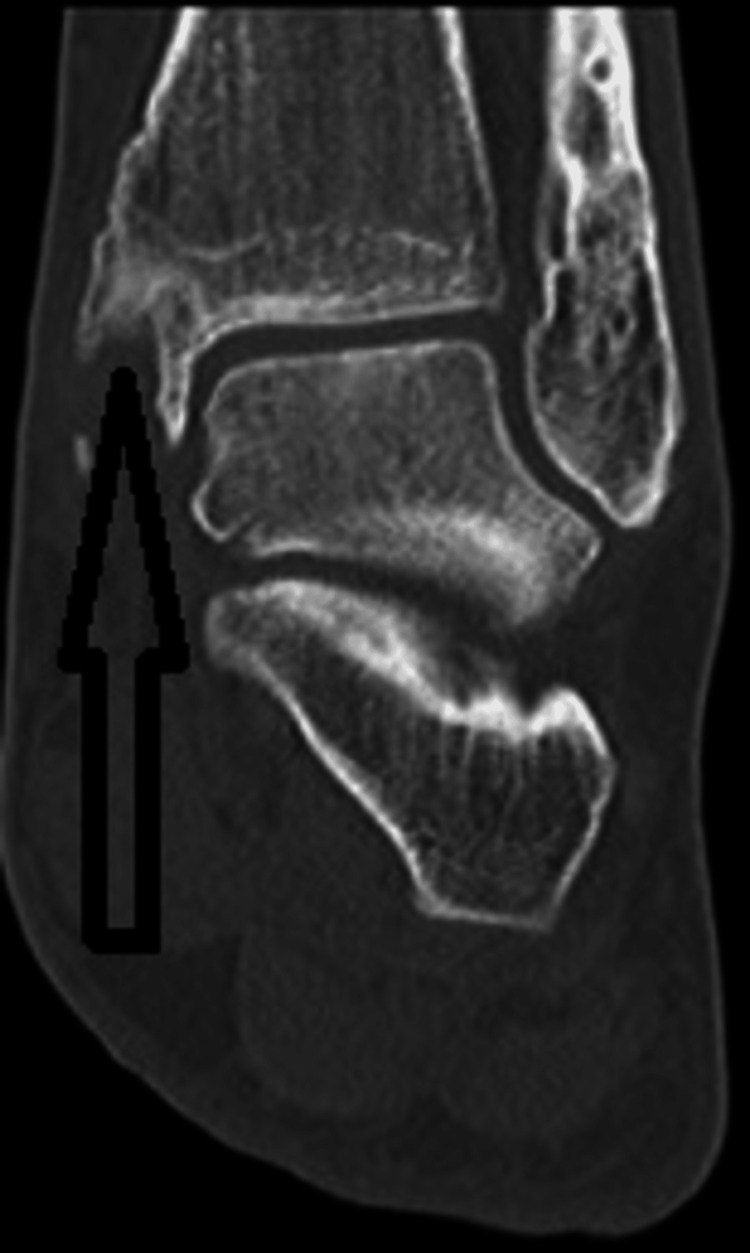
CT scan of the ankle showing the formation of an osseous tunnel in the medial malleolus

**Figure 4:**
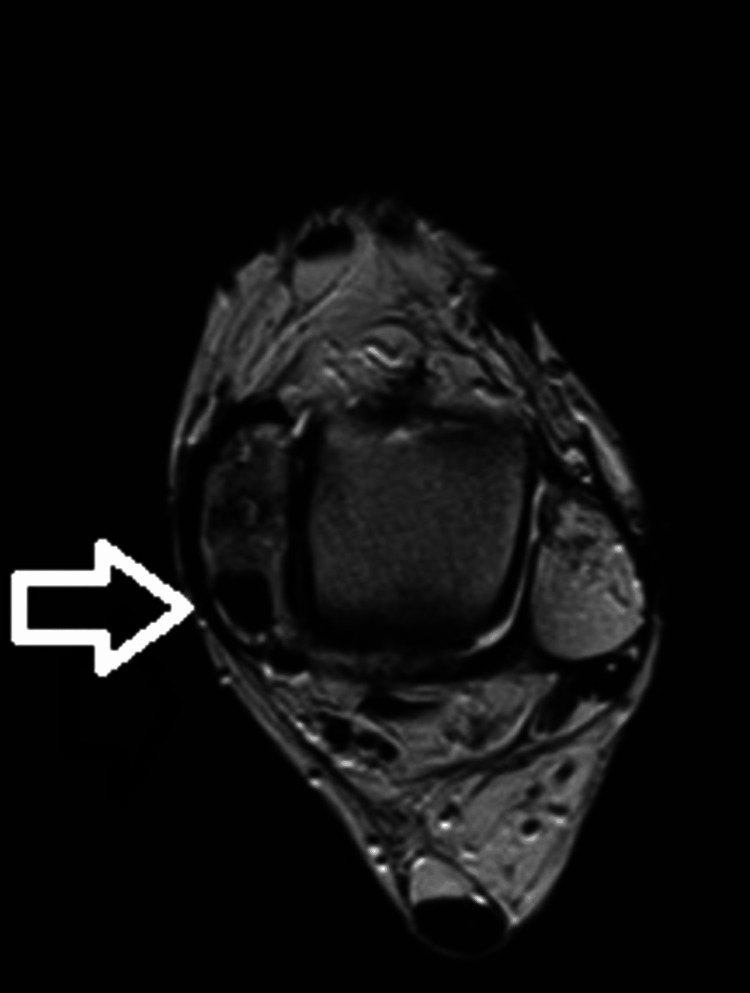
MRI scan of the ankle showing the formation of the osseous tunnel in the medial malleolus

Further imaging, including a CT scan (Figure 3) and an MRI scan (Figure 4), revealed an osseous tunnel encircling the tibialis posterior tendon around the medial malleolus causing stenosing tenosynovitis.

Surgical technique

**Figure 5:**
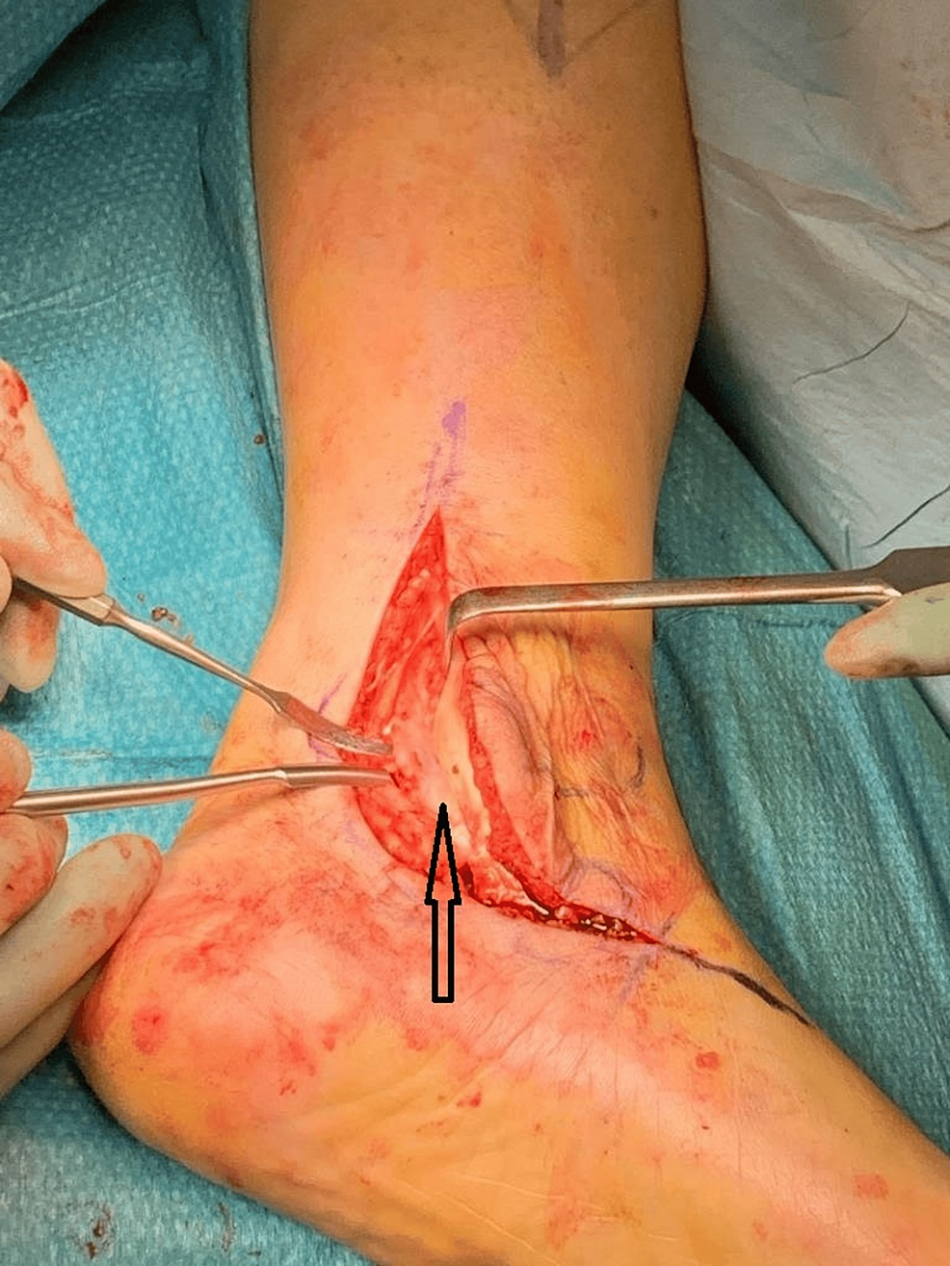
Intraoperative pictures showing the osseous tunnel

**Figure 6:**
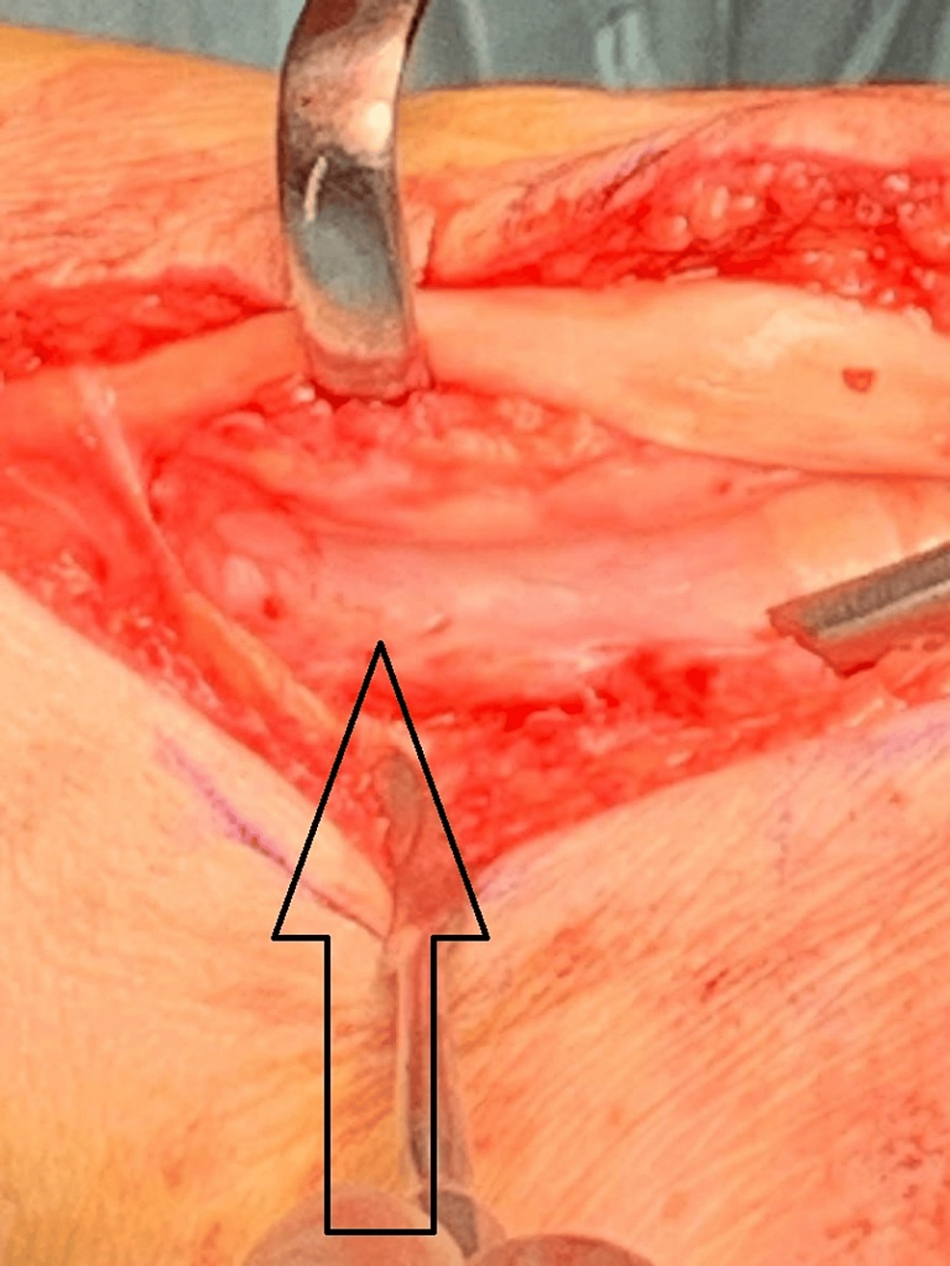
Intraoperative picture showing the floor of the osseous tunnel with the tibialis posterior tendon retracted

A posteromedial approach was employed, involving the incision of the retinaculum at its mid-substance for subsequent repair. The tibialis posterior tendon was meticulously exposed from its distal insertion to its proximal extent in the supra-malleolar region to ascertain the absence of any associated tear or tendinopathy. Intraoperative observations revealed the entrapment of the tendon within the retro-malleolar groove, delineated by post-traumatic heterogeneous ossification. This ossification created a tunnel approximately 3-4 cm in length on either side of the tendon, with the tendon adhering to the tunnel walls while the floor exhibited smoother characteristics (Figures 5-6).

Careful liberation of the tendon and excision of heterogeneous bone ensued, ensuring smooth, unimpeded tendon movement before retinacular repair.

**Figure 7:**
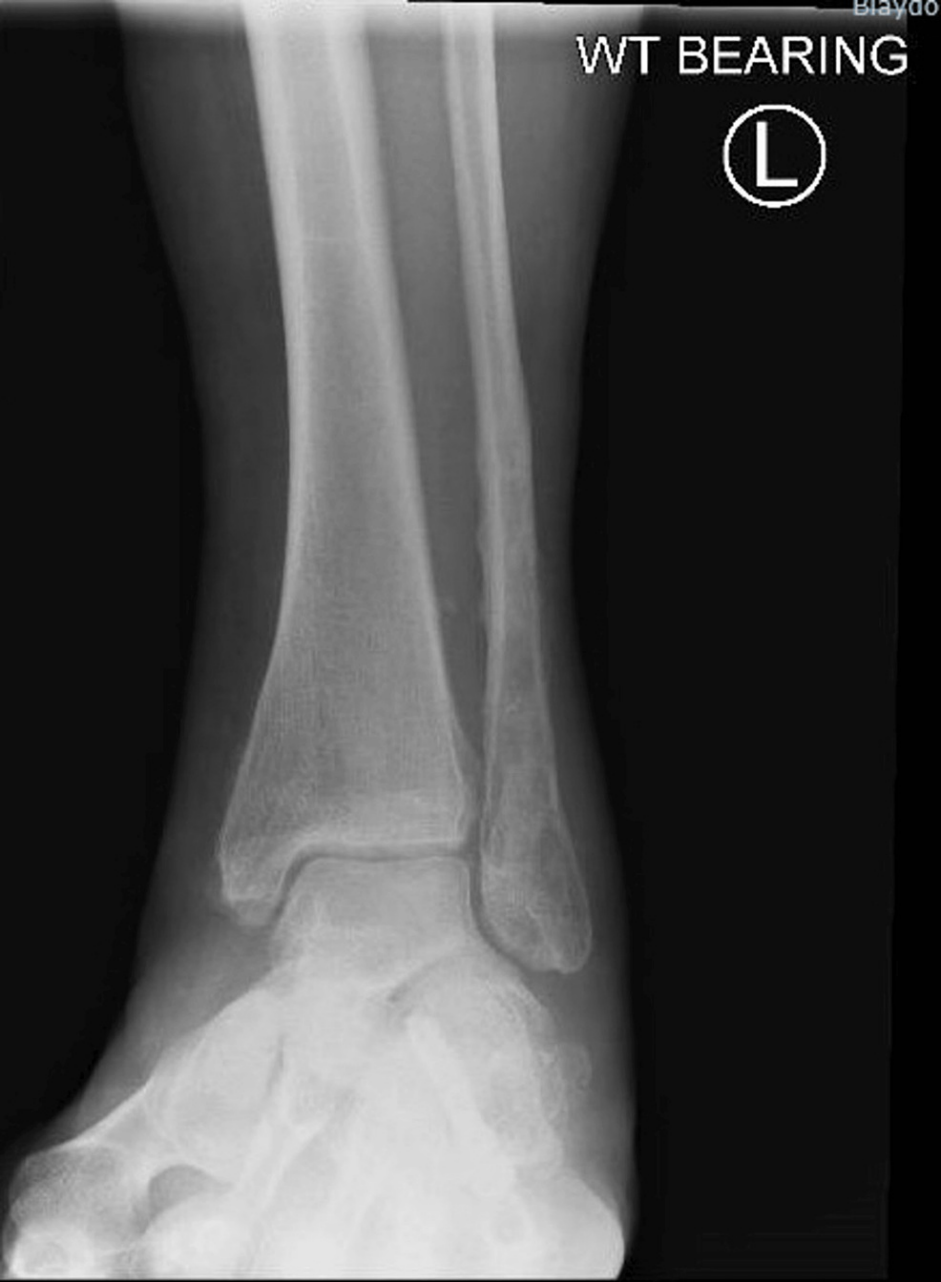
Final AP radiograph showing no bony tunnel around medial malleolus and reconstruction of arch of the foot AP: anteroposterior

**Figure 8:**
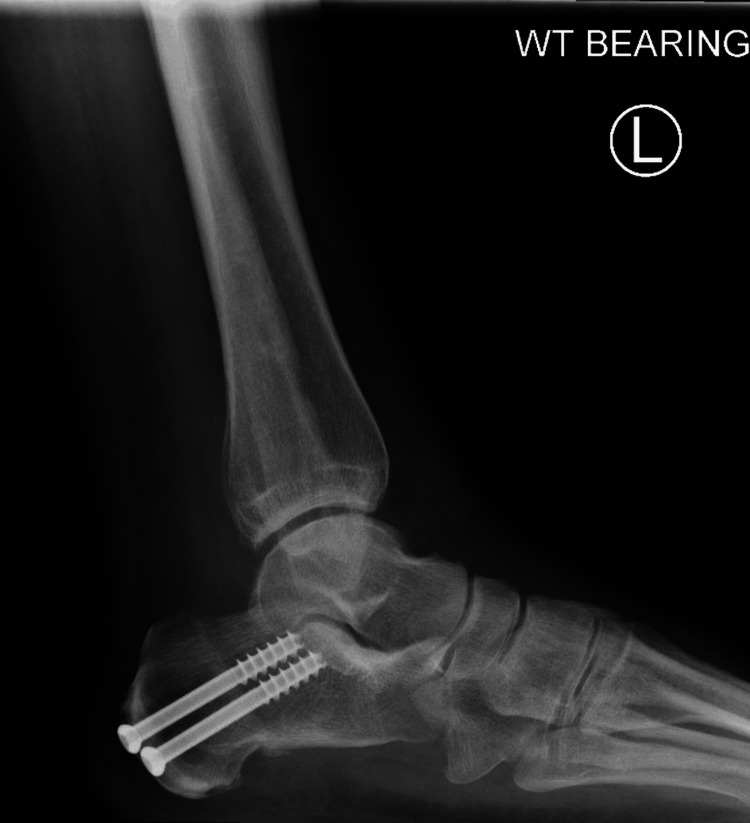
Final lateral radiograph showing no bony tunnel around the medial malleolus and reconstruction of the arch of the foot

The patient then underwent flatfoot reconstruction with calcaneal osteotomy, debridement, excision of osteophytes around the tibialis posterior tendon tunnel, and distalization of the tibialis posterior tendon. The patient was followed up at two weeks for a wound check and received below-knee non-weight-bearing cast treatment every two weeks in a plantigrade position for up to 6 weeks. The patient was given a moon boot and allowed to fully weight-bear for another four weeks. The preoperative American Orthopaedic Foot & Ankle Society (AOFAS) score improved from 39 to 78 at the end of a one-year follow-up. Radiographs taken at the final follow-up show no bony tunnel around the medial malleolus, calcaneal osteotomy well integrated, and good reconstruction of the medial arch (Figures 7-8). 

## Discussion

The tibialis posterior tendon (TPT) is one of the most powerful tendons, located anteriorly in the flexor retinaculum on the medial aspect of the ankle joint [10]. It acts as a plantar flexor and supinator of the foot while maintaining the medial longitudinal arch and stabilizing the subtalar joint [11]. Common pathologies affecting the TPT include chronic tendinopathy, tenosynovitis, enthesopathies, instability, and rupture [12]. Stenosing tenosynovitis is a rare condition affecting the TPT, especially after an ankle fracture.

Tenosynovitis can be divided into three types: peri-tendinitis, chronic tenosynovitis, and stenosing tenosynovitis [13]. Peritendinitis and chronic tenosynovitis usually resolve with conservative treatment, but stenosing tenosynovitis with bone formation usually requires surgical intervention due to tendon entrapment and degeneration within the bony tunnel caused by heterotopic ossification [14].

The radiological investigations of choice for stenosing tenosynovitis are plain X-rays, CT, and MRI scans. However, tenography still plays a role in diagnosis, surgical decision-making, and therapeutic injections of steroids. Tenosynovitis may be graded according to tenography findings, from mild synovitis with minimal irregularity of the sheath to severe synovitis with marked irregularity, outpouchings, nodule formation, and stenosis [15]. In our patient, both CT and MRI scans showed evidence of an extraosseous tunnel formation around the TPT causing stenosing tenosynovitis.

Conservative methods, including modification of activities and footwear, anti-inflammatory medications, orthoses, and injections can be tried, but they are often not successful [16]. Lau et al. described a technique for endoscopic release of the TPT along with synovectomy [17]. Wertheimer et al. claimed good results with the endoscopic approach in treating posterior tibial tenosynovitis resistant to nonsurgical treatment [18].

The treatment of choice is surgical, which includes addressing the tendon itself whether intact, partially ruptured, or completely ruptured, excision of any bony tunnels, deepening the constricted groove, fashioning new pulleys from the available sheath and retinaculum, and constructing a new sheath from the regional deep fascia. Trevino et al., in their series of 12 cases of stenosing tenosynovitis around the ankle (eight posterior tibial and four peroneal), with two to four years of follow-up, reported good results and return to pre-injury activities [2]. In our patient, we excised the bony tunnel, debrided the TPT along with synovectomy, performed a calcaneal osteotomy, and distalization of TPT.

## Conclusions

This case highlights a rare instance of adult-acquired flatfoot resulting from stenosing tenosynovitis of the tibialis posterior tendon with extra-osseous bone formation. Surgical intervention, including the excision of the bony tunnel, debridement, calcaneal osteotomy, and distalization of the tendon, proved effective in alleviating symptoms and restoring function. Early recognition and appropriate surgical management are crucial for preventing progressive deformity and ensuring good functional outcomes in similar cases.
